# Supplementary material for: Action observation intervention using three-dimensional movies improves the usability of hands with distal radius fractures in daily life-A nonrandomized controlled trial in women
Source: PLoS One. 2024 Oct 18;19(10):e0294301. doi: 10.1371/journal.pone.0294301 (PMC11488734; doi:10.1371/journal.pone.0294301)
Supplement: S1 File — (PDF) [file pone.0294301.s001.pdf]

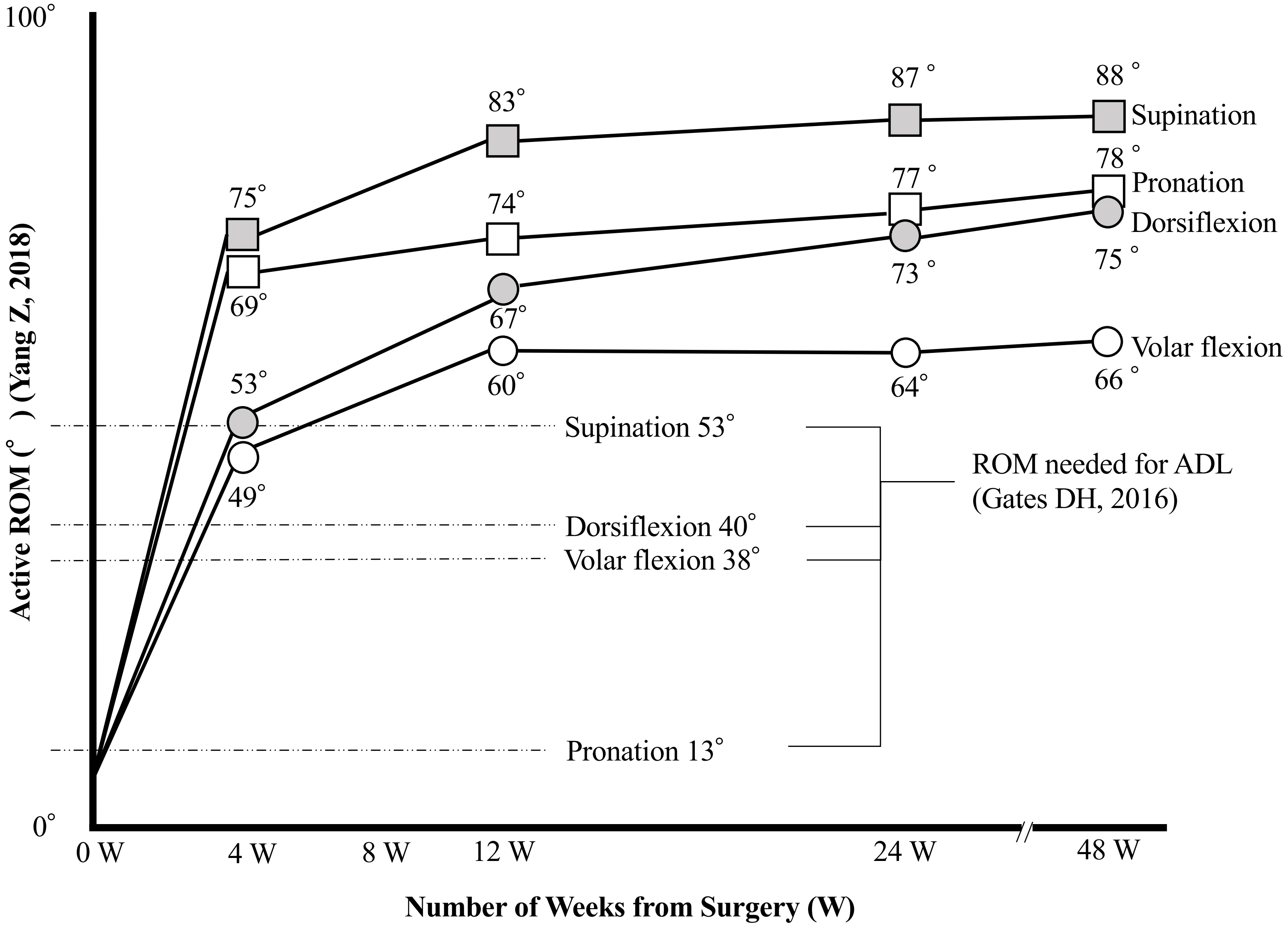

S1. Range of motion of the wrist joint required for activities of daily living.
The minimal angle for each movement is indicated by a dashed line. According to previous literature reports, patients with distal radius fractures achieve that range of motion on average within 1 month after surgery. ADL, activities of daily living; ROM, range of motion; W, week
