# Supplementary material for: Action observation intervention using three-dimensional movies improves the usability of hands with distal radius fractures in daily life-A nonrandomized controlled trial in women
Source: PLoS One. 2024 Oct 18;19(10):e0294301. doi: 10.1371/journal.pone.0294301 (PMC11488734; doi:10.1371/journal.pone.0294301)
Supplement: S2 File — (PDF) [file pone.0294301.s002.pdf]

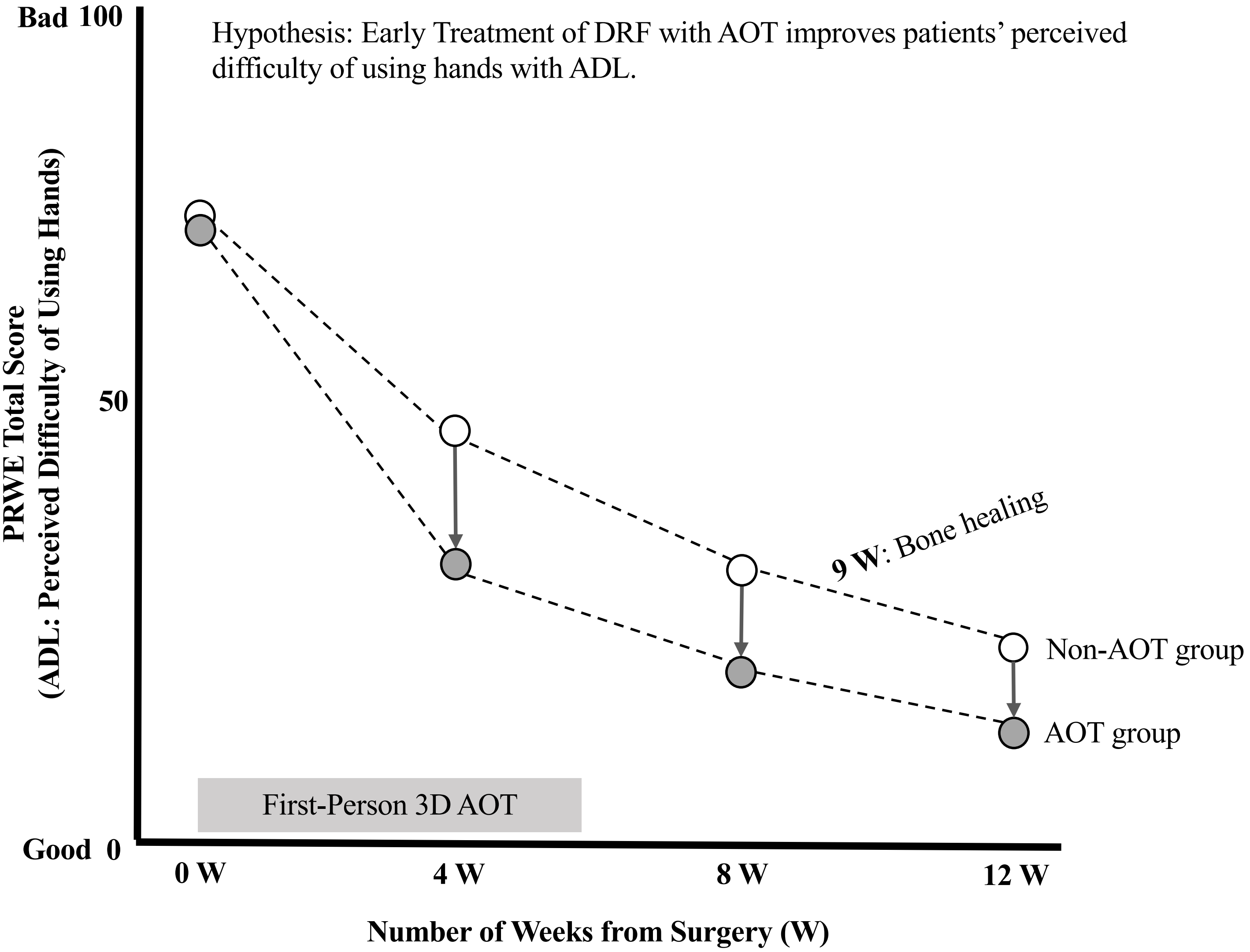

S2. Research hypotheses. AOT in the early postoperative period improves hand use difficulty in patients with DRF from 4 weeks after surgery, when patients are allowed to use their hands. Patient-related Wrist Evaluation (PRWE) scores are shown on the vertical axis. The horizontal axis indicates the time after surgery. ADL, activities of daily living; AOT, action observation therapy; DRF, distal radius fracture; PRWE, Patient-Related Wrist Evaluation; ROM, range of motion; W, week
