# Supplementary material for: Action observation intervention using three-dimensional movies improves the usability of hands with distal radius fractures in daily life-A nonrandomized controlled trial in women
Source: PLoS One. 2024 Oct 18;19(10):e0294301. doi: 10.1371/journal.pone.0294301 (PMC11488734; doi:10.1371/journal.pone.0294301)
Supplement: S3 File — (PDF) [file pone.0294301.s003.pdf]

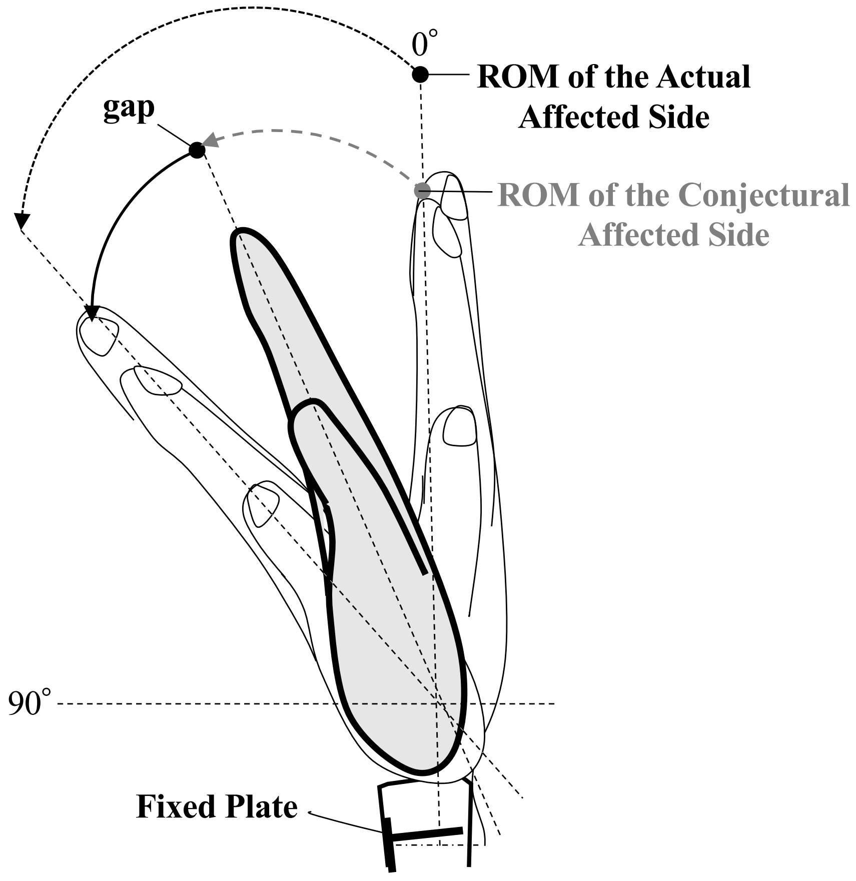

S3. Gap between the estimated and measured ROM of the affected side in wrist flexion. Patients were asked to report the estimated ROM (%) of the affected side when the healthy side was set as 100%. The patient's estimated ROM (%) was converted into the estimated ROM (°) of the affected side, based on the measured ROM (°) of the healthy side. The difference (°) was obtained by subtracting the estimated ROM from the measured ROM of the affected side. ROM, range of motion
