# Supplementary material for: Action observation intervention using three-dimensional movies improves the usability of hands with distal radius fractures in daily life-A nonrandomized controlled trial in women
Source: PLoS One. 2024 Oct 18;19(10):e0294301. doi: 10.1371/journal.pone.0294301 (PMC11488734; doi:10.1371/journal.pone.0294301)
Supplement: S4 File — (PDF) [file pone.0294301.s004.pdf]

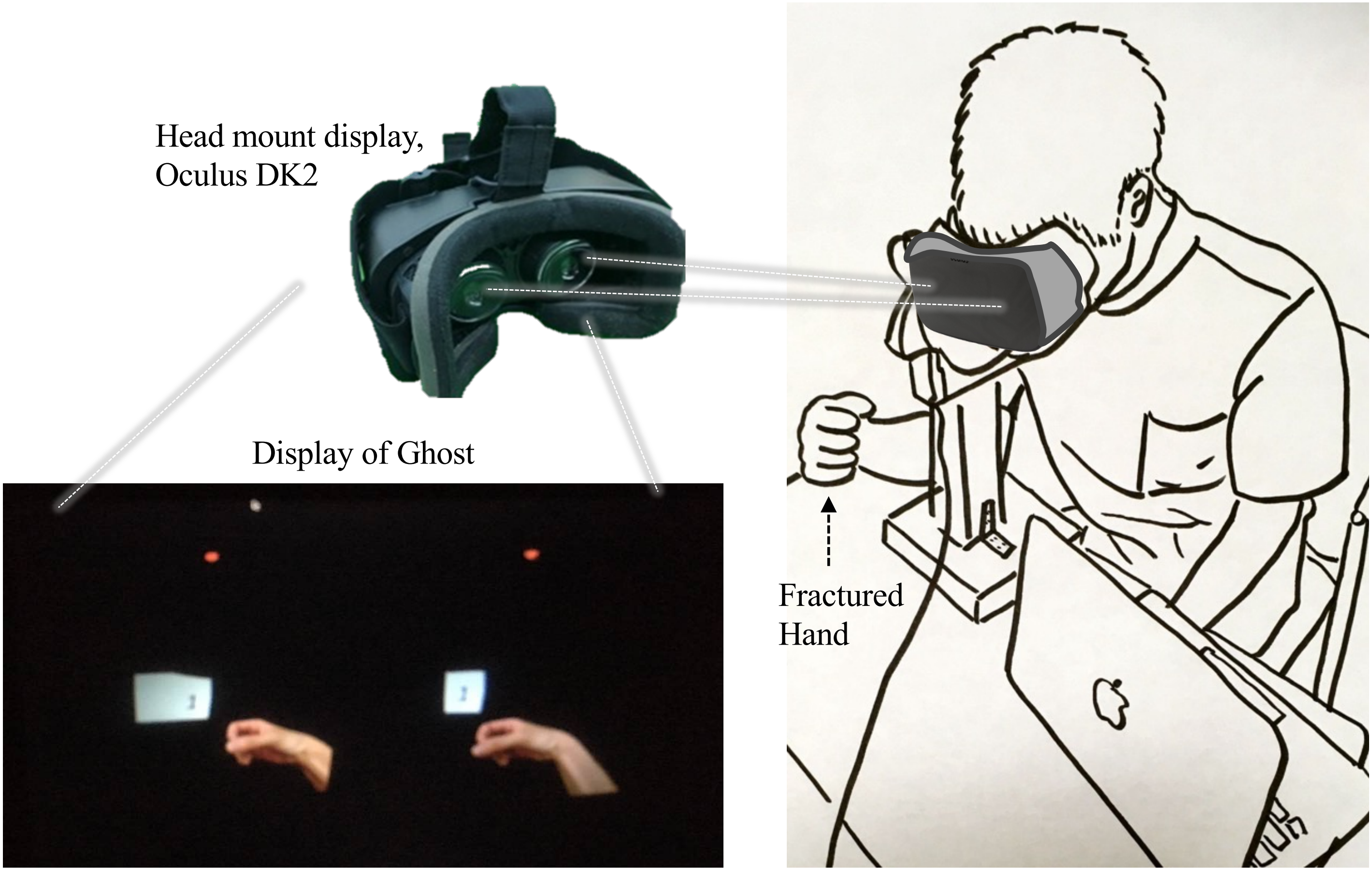

S4. Patient posture during the Ghost intervention. The patient is fitted with an HMD. AOT is performed using first-person 3D images. During the AOT, the patient practices ROM with the fractured hand on the table in accordance with the Ghost video. AOT, action observation therapy; HMD, head-mounted display; ROM, range of motion
