# Supplementary material for: Action observation intervention using three-dimensional movies improves the usability of hands with distal radius fractures in daily life-A nonrandomized controlled trial in women
Source: PLoS One. 2024 Oct 18;19(10):e0294301. doi: 10.1371/journal.pone.0294301 (PMC11488734; doi:10.1371/journal.pone.0294301)
Supplement: S5 File — (PDF) [file pone.0294301.s005.pdf]

研究計画書

「映像観察による運動イメージ課題が橈骨遠位端骨折後の運動予測に与える影響」

１．背景

上肢の骨折による強い疼痛を経験した患者は，骨癒合が確認された後でも，受傷した手を動かすことや負荷をかけることに対して恐怖感や不安を抱くことがある．また，それにより骨折の治癒レベルとしては日常生活で十分に使用できるレベルであるにも関わらず，使用がためらわれるということも多く，日常生活活動（Activity of daily living 以下 ADL）レベルの回復の遅延をもたらす一因にもなっていると考えられる．

Malounin（2009）はヒトの足部関節を固定すると，足部の運動イメージ鮮明度を有意に低下させると報告した．ヒトの大脳は四肢が受傷した時点から痛みのある「動かせない四肢」を学習し始める．これは，固定による四肢の不使用により大脳への入力情報が減少し，大脳皮質の再現領域が縮小するためであると考えられている．大脳皮質の再現領域の再構築により，病的疼痛が誘発されるという説もあり，複合性局所疼痛症候群（以下 CRPS）の要因の一つとして 考えられている（Maihofuner , 2004）．

近年の運動イメージに関する研究結果では，ある運動を脳内でイメージすることにより，実際の運動を伴わなくても，脳内でイメージした運動に対応する大脳皮質の再現領域の活動がみられる（Gallese, 1996, Gazzola，2009）という報告があり，運動イメージは，適切に行われれば一定の再現領域の活動を維持することができる可能性がある．

運動イメージのリハビリテーションへの応用のはじまりは，Ramachandran（1995）による幻肢痛患者に対するミラーセラピーである．ミラーセラピーは，健側を鏡に映して運動を行い，それを観察するものである．患側上肢が運動を行っているかのような視覚的錯覚を生じさせ，それにより生じる運動感覚の入力により，切断によって書き換えられた大脳皮質の再現領域の再構築を促進する．ミラーセラピーはその後，CRPS 患者に対しても試行され，同様に疼痛を軽減する効果があると報告された（Moseley, 2004）．さらには脳卒中片麻痺患者に対しても応用が試みられ，手指機能の改善が報告されている．

運動イメージのリハビリテーションにおける応用は，映像を用いた運動観察という手段によっても行われており，Ertelt（2007）は維持期の脳卒中患者を対象に，上肢の 54 種類の目的動作の映像観察と運動の反復練習を実施し，上肢機能の改善を報告している．パーキンソン病の患者を対象とした研究では，映像による運動観察と運動イメージを組み合わせることにより，大脳皮質運動野の可塑的変化をもたらし，運動パフォーマンスの改善を誘発することが可能であったとしている（Abbruzzese，2015）．

運動観察と運動イメージに関する研究は目覚ましく進んでいるものの，これらを組み合わせたリハビリテーションプログラムとして系統立てられたものは，Moseley（2004）による CRPS 患者を対象としたプログラムが代表的であるが，それ以降も主に，CRPS や慢性疼痛を対象とした運動イメージのプログラムの報告がある（Priganc, 2011）．脳卒中および整形外科疾患に対するリハビリテーションにおいては，運動イメージを治療経過の中の一部のみに対して実施したものである．しかし，整形外科疾患に対して，受傷直後から一定期間を対象とした系統立てられた運動イメージのリハビリテーションプログラムに関する報告は見当たらない．

映像を用いた運動観察の方法としては，これまで液晶モニターで目的の運動を見せる，というものがほとんどであった．また，映像を一人称的視点から見せるのか，三人称的視点から見せるのかという点にお

いての議論があり，一人称的視点から観察した方が 3 人称的視点から観察した時よりも，感覚運動皮質での脳活動量が多いという報告（Philip，2006）がある．一人称的視点の特徴は，あたかも自分自身が映像の中の運動を行っているような視点で運動を再現しているという点であり，つまり，映像の中の手をよりリアルに「自分の手」と認識または錯覚できるような見せ方が重要であるといえる．

Oculus Rift は，ゴーグル型のモニターであるが，右目用レンズと左目用レンズがあり，左右それぞれに対応した視差画像を投影することにより，視差を利用した立体視が可能になるものである．加えて，ゴーグル装着中の視野においては，投影される映像のみがある状態となり，周辺の余計な背景が視界に入ることはなく，映像に集中しやすい環境を作り出すことが可能である．このような映像観察の環境によって，一人称的視点の利点を強調することができると考える．

よりリアルな運動イメージを，上肢の骨折後の機能練習プログラムに導入することにより，手本とすべき運動のイメージを持ってもらうことが可能となり，受傷前に動いていた自分の手のイメージを縮小することなく，つまりは，受傷直後から始まる大脳と末梢の障害部の変化をできるだけ早期から防止し，大脳皮質の再現領域の維持を図り，それによって，運動の再学習をより早く促すことができるのではないかと考えた．

２．目的

運動イメージ練習プログラムを上肢の骨折（橈骨遠位端骨折）に対する一般的な作業療法治療プログラムに加えて実施し，効果を検証する

３．対象

１）橈骨遠位端骨折患者

（１）保存療法の患者（TFCC 損傷，尺骨損傷含む）

（２）観血的治療の患者（ORIF），（TFCC 損傷，尺骨茎状突起損傷含む）

２）女性

３）右利き

４）年齢 70 歳以下

５）中枢神経疾患を有さず，コミュニケーション障害がなく，認知機能が良好な成人

上記の１）～５）を満たす者を，映像観察群と，対照群に割り付ける．

４．方法

１）実施場所

北里大学メディカルセンター　リハビリテーションセンター

２）道具

ノートパソコン１台（アプリケーションインストール済み），バスタオル，Oculus Rift

３）データの収集方法および測定用具

（１）対象者の基本属性

性別，年齢，骨折分類（AO 法），患手，受傷機転，手術日，骨アライメント，リハビリテーション開始日，利き手をカルテから収集する

（２）運動機能

ア．関節可動域

イ．握力（JAMER 握力計）

ウ．巧緻動作（Purdue Peg Board）

（３）自己予測運動と実際の運動の比較

イ．患側関節角度予測

健側可動域を測定後，患側が健側比何%程度，掌屈・背屈・回内・回外できるか予測させる．健側で再現させる．

（４）日常生活動作，疼痛，および QOL

ア．Disability Arm Shoulder and Hand（日本語版）

イ．Patient Related Wrist Evaluation（日本語版）

ウ．Mayo Wrist Score

エ．自由記載：患者が困難を感じる動作を聞き取り調査する．

オ．タオル絞り：水に濡らしたタオルを絞らせて重量を測定する．

４）映像観察について

（１）実施時期

毎回の作業療法実施後の自動関節可動域訓練時に実施する（自主トレーニングには組み込まない）

（３）実施頻度

1 回/週　以上

（４）実施期間

受傷後 0 週～12 週

５）映像の構成（右手用，左手用で同内容）

（１）手指の[屈曲（1 秒），伸展（1 秒）]　10 回　（20 秒）

（２）4 秒休憩

（３）手関節の[背屈（1 秒），掌屈（1 秒）]　10 回　（20 秒）

（４）手関節の[背屈（4 秒），掌屈（4 秒）]　5 回　（40 秒）

※（３），（４）を 5 回繰り返す

（５）8 秒休憩

（６）前腕の[回外（1 秒），回内（1 秒）]　10 回　（20 秒）

（７）前腕の[回外（4 秒），回内（4 秒）]　5 回　（40 秒）

※（６），（７）を 5 回繰り返す

※（１）～（７）の全てを視ると 10 分 32 秒かかる

５．手順

１）倫理的手続き

本研究は北里大学メディカルセンター倫理委員会，埼玉県立大学倫理委員会の承認を得て実施する．

2）対象者への研究の説明と同意

主治医からリハビリテーション依頼のあった橈骨遠位端骨折患者を対象とし，リハビリテーションセンターに来室した際に担当者の紹介を行う．研究責任者が説明文書を用いて研究の対象，目的，方法，研究参加による利益・不利益と倫理的配慮を口頭で説明し，対象者の理解を得る．その後，同意書に署名をいただき書面による同意を得る．被験者が研究協力を取り消すときは口頭または書面によって行う．

3）映像観察の実施

映像観察群：Oculus Rift を装着し，映像を観察し，映像に合わせて自身の手指，手関節，前腕の自動運動を行う

対照群：作業療法における通常の自動関節可動域訓練を実施する

かかる時間；10 分程度（実施者の準備時間除く）

自動運動の程度：医師の許可に基づき，各患者および治癒過程に応じた，無理のない範囲で運動を行う．

4）対象者の評価測定の実施

（1）初回のリハビリテーションセンター来室時に測定内容や所要時間を説明して測定を実施する．

（2）測定はリハビリテーション開始の初回，1 カ月，2 カ月，3 カ月の 4 回測定とする．

（3）測定時間は合計 40 分とし，測定中は適宜休憩をしながら実施する．

5）解析方法

介入群と未介入群にわけ 1 か月時点での自己予測運動と実測運動との間に介入時にノンパラメトリック検定. 3 か月間を 2 元配置の分散分析にて評価を実施する.

6．中止基準

1）中止の申し出があった場合

2）一般的なリハビリテーション中止基準

3）映像を観察することによる気分不快，体調不良，疼痛その他の症状の出現があった場合

4）その他，実験実施者が中止した方が良いと判断した場合

7．倫理的配慮

1）研究参加の自由

（1）対象者には, 主治医の了解を得て研究責任者が研究説明書を用いて口頭で説明する. その後, 書面による同意を得る.

（2）研究参加は対象者の自由意思により決定され, いつでも承諾の撤回が可能である. そして, 途中で測定を辞退した場合でも, 辞退したことによる診療への影響は全くないことを約束する.

2）測定の実施における配慮

（1）測定方法は対象者の負担とならない状況で実施することを最優先し, 対象者の体調に配慮し, 安全を確保し不要なストレスを与えないようにする.

（2）測定時間はあくまでも目安であり，対象者が測定を終了したい意志がある場合には，直ちに中止する．また，対象者の過度な負担とならないよう時間の調節を行う．

3）個人情報の保護

（1）対象者の個人情報を保護するため，検査中は仕切りなどで他者から見えない場所で実施する．

（2）研究により得られた情報は本研究のみに使用し，対象者の許可なく他者には伝えない．

（3）データの管理はコード番号等で行い，氏名など個人情報が特定できないようにする．

（4）対象者の個人情報は北里大学メディカルセンターの倫理規定に従い，第三者の立ち入りを制限した環境下で測定を実施し，情報流出を防ぐために徹底した管理を行う．

（5）本研究にて得られた結果は学会や論文にて発表する．ただし，発表の際には個人が特定できる情報を一切含まない．

4）不測の事態発生時の対応

（1）研究責任者は測定開始時に，対象者に対して体調を確認し，心身状態をよく観察する．対象者の体調がすぐれない際には測定は中止し，担当主治医に報告する．

（2）測定中も対象者の状態の変化の有無を常に観察し，対象者に対しては体調がすぐれない場合にはいつでも申しでるよう伝え，体調が悪化した場合には測定を即刻中止し，担当主治医に報告する．

8．参考文献

1）Malouin, Francine F：Effects of practice, visual loss, limb amputation, and disuse on motor imagery vividness. Neurorehabilitation and neural repair 23(5): 449-463, 2009

2）Maihöfner C1, Handwerker HO, Neundörfer B, Birklein F：Cortical reorganization during recovery from complex regional pain syndrome. Neurology 63(4): 693-701, 2004

3）Vittorio Gallese, Luciano Fadiga, Leonardo Fogassi, Giacomo Rizzolatti：Action recognition in the premotor cortex. Brain 119: 563-609, 1996

4）Valeria Gazzola, Christian Keysers：The observation and execution of actions share motor and somatosensory voxels in all tested subjects: single-subject analysis of unsomoothes fMRI data. cerebral cortex 19：1239-1255,2009

5）V. S. Ramachandran, D.Rogers-Ramachandran, S.Cobb：Touching the phantom limb. Nature 377：489-490, 1995

6）Moseley GL：Graded motor imagery is effective for long-standing complex regional pain syndrome: a randomised controlled trial. Pain 108(1-2), 192-198, 2004

7）Victoria W. Priganc, Susan W. Stralka：Graded motor imagery. Journal of hand therapy24（2）164-169, 2011

8）Denis Ertelt, Steven Small, Ana Solodkin, Christian Dettmers, Adam McNamara, Ferdinand Binkofski, Giovanni Buccino：Action observation has positive impact on rehabilitation of motor deficits after stroke. Neuroimage 36：T164-T173, 2007

９）Giovanni Abbruzzese, Laura Avanzino, Roberta Marchese, Elisa Pelosin：Action observation and motor imagery: innovative cognitive tools the rehabilitation of parkinson's disease. Parkinson's disease 2015：1-9,2015
１０）Philip L.Jacson, Andrew N. Meltzoff, Lean Decety：Neural circuits involved in imitation and perspective-taking. NeuroImage 31：429-439, 2006

９．研究代表者

埼玉県立大学大学院保健医療福祉学研究科
北里大学メディカルセンター　リハビリテーションセンター　作業療法士　薄木　健吾

１０．連絡先・問い合わせ

北里大学メディカルセンター　リハビリテーションセンター　作業療法士　薄木　健吾
埼玉県北本市荒井 6 丁目 100 番地　電話番号　048－593－1212

Research Proposal

"Influence of Motor Imagery Tasks through Video Observation on Motor Prediction after Distal Radius Fracture"

Background

Patients who have experienced strong pain due to upper limb fractures may harbor fear and anxiety about moving or loading the injured hand even after confirming bone healing. This fear can persist, leading to hesitation in using the injured hand in daily activities, potentially causing a delay in the recovery of Activities of Daily Living (ADL) levels, despite the fracture healing to a level sufficient for daily life use. Malounin (2009) reported a significant decrease in the clarity of foot joint movement imagery when fixing the human foot joint. The human brain begins to learn the "immobile limb" with pain from the moment the limbs are injured. This is believed to be due to the reduction of input information to the brain caused by limb non-use due to fixation, leading to a shrinkage of the representation area in the cerebral cortex. The reconstruction of the representation area in the cerebral cortex is also suggested to induce pathological pain and is considered one of the factors for Complex Regional Pain Syndrome (CRPS) (Maihofuner, 2004).

Recent research on motor imagery suggests that imagining a movement in the brain activates the corresponding representation area in the cerebral cortex, even without actual movement (Gallese, 1996; Gazzola, 2009). If done correctly, motor imagery has the potential to maintain activity in certain representation areas. The application of motor imagery to rehabilitation began with Ramachandran's (1995) mirror therapy for patients with phantom limb pain. Mirror therapy involves performing movements while observing the reflected image in a mirror, creating a visual illusion that the affected limb is moving. This visual feedback promotes the reconstruction of the representation area in the cerebral cortex that was altered by amputation. Mirror therapy has been tried for CRPS patients, showing a pain-reducing effect (Moseley, 2004). It has also been applied to stroke patients, resulting in reported improvements in hand function.

The use of motor imagery in rehabilitation has also been implemented through video-based movement observation. Ertelt (2007) conducted a study with chronic stroke patients, combining video observation of 54 upper limb movements with repetitive exercises, reporting improved upper limb function. In research involving Parkinson's disease patients, combining video-based movement observation and motor imagery induced plastic changes in the primary motor cortex and improved motor performance (Abbruzzese, 2015).

While research on movement observation and motor imagery has progressed significantly, organized rehabilitation programs combining these elements are limited. Notable among them is Moseley's (2004) program for CRPS patients. Since then, there have been reports on programs focusing mainly on motor imagery for CRPS and chronic pain (Priganc, 2011). In rehabilitation for stroke and orthopedic conditions, motor imagery has been applied to specific aspects of the treatment process. However, there is a lack of systematic reports on motor imagery rehabilitation programs targeting orthopedic conditions, specifically from the early stages of injury.

In the context of video-based movement observation, the predominant method has been to display the desired movement on a liquid crystal display (LCD) monitor. There has been debate about whether to present the video from a first-person perspective or a third-person perspective. There is a report suggesting that observing from a first-person perspective results in more significant brain activity in the sensorimotor cortex than observing from a third-person perspective (Philip, 2006). The characteristic of a first-person perspective is the reproduction of

movement from the viewpoint as if the observer is performing the movement, allowing for a more realistic recognition or illusion of the hand in the video as "one's own hand."

Oculus Rift is a goggle-type monitor with separate lenses for the right and left eyes, allowing for stereoscopic vision by projecting disparate images for each eye. Additionally, while wearing the goggles, only the projected images are visible in the field of view, creating an environment where peripheral background distractions are eliminated, facilitating concentration on the video. It is believed that such an environment for video observation can emphasize the advantages of a first-person perspective.

By introducing a more realistic motor imagery into the functional exercise program for upper limb fractures, it is possible to provide patients with an image of the movement to be emulated, maintaining the image of their own hand in motion from before the injury. In other words, by preventing changes in the damaged areas of the brain and peripheral nerves as early as possible, starting from immediately after the injury, maintaining the representation area in the cerebral cortex, it may be possible to promote faster relearning of movement.

Objectives
To implement a motor imagery training program in addition to a conventional occupational therapy program for upper limb fractures (distal radius fractures) and verify its effectiveness.

Participants

Patients with distal radius fractures
(1) Patients undergoing conservative treatment (including TFCC injury, ulnar bone injury)
(2) Patients undergoing surgical treatment (ORIF) (including TFCC injury, ulnar styloid process injury)
Female
Right-handed
Age under 70
Adults with no central nervous system disorders, no communication disorders, and good cognitive function
Participants meeting the criteria of 1) - 5) will be assigned to the video observation group and the control group.
Methods
Implementation Location
Keio University Medical Center Rehabilitation Center

Tools
One laptop (with installed application), bath towel, Oculus Rift

Data Collection Methods and Measurement Tools
(1) Basic attributes of subjects
Gender, age, fracture classification (AO method), injured hand, mechanism of injury, surgery date, bone alignment, rehabilitation start date, dominant hand, collected from medical records
(2) Motor function
A. Joint range of motion

B. Grip strength (JAMER grip meter)
C. Fine motor skills (Purdue Peg Board)
(3) Comparison of self-predicted and actual movements
B. Prediction of the joint angle on the affected side
After measuring the range of motion on the healthy side, predict the percentage of how much the affected side can perform flexion, extension, pronation, and supination. Reproduce on the healthy side.
(4) Daily life activities, pain, and QOL
A. Disability Arm Shoulder and Hand (Japanese version)
B. Patient-Related Wrist Evaluation (Japanese version)
C. Mayo Wrist Score
D. Free description: Survey to inquire about movements that the patient finds difficult.
E. Towel wringing: Measure the weight by squeezing a wet towel.

Video Observation
(1) Implementation timing
Conducted during every occupational therapy session after automatic joint range of motion training (not included in self-training).
(3) Implementation frequency
Once a week or more
(4) Implementation period
From 0 weeks to 12 weeks post-injury

Composition of the video (same content for right and left hands)
(1) Finger flexion (1 second), extension (1 second) - 10 times (20 seconds)
(2) 4-second break
(3) Wrist dorsiflexion (1 second), palmar flexion (1 second) - 10 times (20 seconds)
(4) Wrist dorsiflexion (4 seconds), palmar flexion (4 seconds)

5. Procedures

Ethical Procedures
This study will be conducted with the approval of the Ethics Committees of Kitasato University Medical Center and Saitama Prefectural University.

Explanation and Consent to Participants
Patients with distal radius fractures who have received a rehabilitation request from their attending physician will be introduced to the study when they visit the Rehabilitation Center. The principal investigator will orally explain the target, purpose, method, benefits/disadvantages of participating in the study, and ethical considerations using an explanatory document. Subsequently, the participant's understanding will be obtained, and written consent will be obtained through the signing of a consent form. If a participant wishes to withdraw from the study, this can be done orally or in writing.

Implementation of Video Observation
Video Observation Group: Wearing Oculus Rift, participants will observe the video and perform automatic movements of their fingers, hand joints, and forearms in sync with the video.

Control Group: The usual automatic joint range of motion training in occupational therapy will be implemented.

The time required is approximately 10 minutes (excluding preparation time for the implementer). The degree of automatic movement will be determined based on the physician's permission, and participants will perform movements within a reasonable range according to each patient and healing process.

Implementation of Participant Evaluation Measurement
(1) Explain and conduct measurements of the content and required time when the participant first visits the Rehabilitation Center.

(2) Measurements will be conducted four times: at the first rehabilitation session, 1 month, 2 months, and 3 months after the start of rehabilitation.

(3) The total measurement time is 40 minutes, and measurements will be carried out with breaks as needed.

5) Analysis Method

Separate the intervention and non-intervention groups and evaluate the non-parametric test at the 1-month point and the two-way analysis of variance over the 3-month period between the predicted self-movement and actual movement at the time of intervention.

6. Criteria for Discontinuation

If there is a request for discontinuation.

General rehabilitation discontinuation criteria.

If there is discomfort, physical discomfort, pain, or other symptoms due to observing the video.

In other cases where the experimenter judges that it is better to discontinue.

7. Ethical Considerations

Freedom to Participate in the Study
(1) Participants will be orally explained using a research explanatory document with the understanding of the attending physician. Afterward, written consent will be obtained.

(2) Participation in the study is determined by the free will of the participants and can be withdrawn at any time.

Even if the measurement is declined midway, it is promised that there will be no impact on medical care.

Consideration for Measurement Implementation

(1) The measurement method will prioritize being conducted in a situation that does not burden the participants. Care will be taken for the participants' physical condition, ensuring safety, and avoiding unnecessary stress.

(2) The measurement time is only a rough guide, and if participants wish to end the measurement, it will be stopped immediately. Also, adjustments will be made to the time to avoid excessive burden on the participants.

Protection of Personal Information

(1) The measurement will be conducted in a place where others cannot see, using partitions, etc., to protect the personal information of the participants.

(2) Information obtained from the study will be used only for this research and will not be disclosed to others without the participant's permission.

(3) Data management will be conducted with code numbers, etc., to ensure that personal information cannot be identified.

(4) The personal information of the participants will be managed in accordance with the ethical regulations of Kitasato University Medical Center, and strict management will be carried out in an environment that restricts third-party access to prevent information leakage.

(5) Results obtained from this study will be presented at conferences and in papers. However, personal information that can identify individuals will not be included in any presentations.

Response to Unforeseen Events

(1) At the start of measurement, the principal investigator will confirm the participant's physical condition and observe their mental and physical state. If the participant's condition is not good, the measurement will be discontinued, and the attending physician will be informed.

(2) During measurement, the participant's state will be constantly observed. The participant will be informed to report immediately if they feel unwell, and if their condition worsens, the measurement will be immediately stopped, and the attending physician will be informed.

8. References

Malouin, F., et al. (2009). Effects of practice, visual loss, limb amputation, and disuse on motor imagery vividness. Neurorehabilitation and neural repair, 23(5), 449-463.

Maihöfner, C., et al. (2004). Cortical reorganization during recovery from complex regional pain syndrome. Neurology, 63(4), 693-701.

Gallese, V., et al. (1996). Action recognition in the premotor cortex. Brain, 119, 563-609.

Gazzola, V., et al. (2009). The observation and execution of actions share motor and somatosensory voxels in all tested subjects: single-subject analysis of unsmoothed fMRI data. Cerebral cortex, 19, 1239-1255.

Ramachandran, V. S., et al. (1995). Touching the phantom limb. Nature, 377, 489-490.

Moseley, G. L. (2004). Graded motor imagery is effective for long-standing complex regional pain syndrome: a randomized controlled trial. Pain, 108(1-2), 192-198.

Priganc, V. W., et al. (2011). Graded motor imagery. Journal of hand therapy, 24(2), 164-169.

Ertelt, D., et al. (2007). Action observation has a positive impact on rehabilitation of motor deficits after stroke. Neuroimage, 36, T164-T173.

Abbruzzese, G., et al. (2015). Action observation and motor imagery: innovative cognitive tools for the rehabilitation of Parkinson's disease. Parkinson's disease, 2015, 1-9.

Jacson, P. L., et al. (2006). Neural circuits involved in imitation and perspective-taking. NeuroImage, 31, 429-439.

9. Principal Investigator

Saitama Prefectural University Graduate School of Health Care and Welfare Kitasato University Medical Center Rehabilitation Center Occupational Therapist, Kengo Usuki

10. Contact Information

Kitasato University Medical Center Rehabilitation Center Occupational Therapist, Kengo Usuki 6-100 Arai, Kitamoto City, Saitama Phone Number: 048-593-1212
