# Supplementary material for: Action observation intervention using three-dimensional movies improves the usability of hands with distal radius fractures in daily life-A nonrandomized controlled trial in women
Source: PLoS One. 2024 Oct 18;19(10):e0294301. doi: 10.1371/journal.pone.0294301 (PMC11488734; doi:10.1371/journal.pone.0294301)
Supplement: Attachment — Submitted filename: renamed_f3520.docx [file pone.0294301.s008.docx]

# COMMENTS – Manuscript PONE-D-23-33804

Title: “Action observation intervention using three-dimensional movies improves the usability of hands with distal radius fractures in daily life: a nonrandomized controlled trial in women”

**Important note: This review pertains only to ‘statistical aspects’ of the study and so ‘clinical aspects’ [like medical importance, relevance of the study, ‘clinical significance and implication(s)’ of the whole study, etc.] are to be evaluated [should be assessed] separately/independently. Further please note that any ‘statistical review’ is generally done under the assumption that (such) study specific methodological [as well as execution] issues are perfectly taken care of by the investigator(s). This review is not an exception to that and so does not cover clinical aspects {however, seldom comments are made only if those issues are intimately / scientifically related & intermingle with ‘statistical aspects’ of the study}. Agreed that ‘statistical methods’ are used as just tools here, however, they are vital part of methodology [and so should be given due importance]. I look at the manuscript in/with statistical view point, other reviewer(s) look(s) at it with different angle so that in totality the review is very comprehensive. However, there should be efforts from authors side to improve (may be by taking clues from reviewer’s comments). Therefore, please do not limit the revision only (with respect) to comments made here.**

**COMMENTS:** I noted that your ABSTRACT is well drafted (in my opinion), but is ‘assay type’. It is preferable [refer to item 1b of CONSORT checklist 2010: Structured summary of trial design, methods, results, and conclusions] to divide the ABSTRACT with small sections like ‘Objective(s)’, ‘Methods’, ‘Results’, ‘Conclusions’, etc. which is an accepted practice of most of the good/standard journals [including this one, though ‘The PLoS One Guidelines to Authors’ did not specify an Abstract format, it is desirable]. It will definitely be more informative then, I guess, whatever the article type may be [since the ‘Article Type’ is ‘Clinical Trial’ it is necessary indeed].

There are more issues (a few serious) about which I have different opinion. Such observations/concerns are given below:

This trial/study is on very important topic but title of the study [“**Action observation intervention using three-dimensional movies improves the usability of hands with distal radius fractures in daily life**”] is little confusing. It gives the impression that authors are interested [their main focus is on] in ‘three-dimensional video during ROM exercises’ whereas in lines 25-28 it is clearly mentioned that ‘This nonrandomized controlled trial tested the hypothesis that AOT improves hand-use difficulties during activities of daily living in patients with DRF’. Action observation intervention is the main intervention [lines 30-31: The primary outcome was the difficulty in using the fractured hand and line 27: AOT improves hand-use difficulties] which is evaluated but administered using three-dimensional movies. You may think of changing the title.

According to table-2 on page 158 of Jacob Cohen’s paper “A power primer” in Psychological Bulletin, 1992, vol.:112, pp 155-159 [which is a sort of summary of the excellent book by Cohen himself titled ‘Statistical power analysis for the behavioral sciences’, Academic Press, 1977, New York] even for medium effect size you need n=64 per group (type-I error=0.05, power=80%). This is given here in the context of account in lines 335-36 [where you said: “the final target sample size was set to be 34 cases in total, 17 cases in each group”]. Although you have quoted the reference number 28 { Schott N, Korbus H. Preventing functional loss during immobilization after osteoporotic wrist fractures in elderly patients : a randomized clinical trial. BMC Musculoskelet Disord. 2014; 15: 287 but this study is a randomized with three intervention groups & as I understand, it is only a protocol} please check the ‘control’ group to be used and all other issues. Was it (study described in reference 28) simillar [with respect to ‘design’] to yours?

Since (lines 340-41) “The primary outcome of this study was the PRWE score as a measure of the difficulty in using hands for ADLs” please check the level of measurement of data (to be) yielded by ‘The PRWE scale/questionnaire’. Though this measure/tool used is appropriate, is likely to yield data that are in [at the most] ‘ordinal’ level of measurement [and not in ratio level of measurement for sure {as the score two times higher does not indicate presence of that parameter/phenomenon as double (for example, a Visual Analogue Scales VAS score or say ‘depression’ score)}].

Then application of suitable non-parametric (or distribution free) test(s) is/are indicated/advisable [even if distribution may be ‘Gaussian’ (also called ‘normal’)]. Agreed that there is/are no non-parametric test(s)/technique(s) available to be used as alternative in all situation(s), but should be used whenever/wherever they are available. Therefore, in short use suitable non-parametric test(s)/technique(s) while dealing with data that are in ‘ordinal’ level of measurement even if [despite that] the distribution may be ‘Gaussian’.

This is pasted from one famous standard textbook on ‘Medical Research Methodology’ and authors may think of using ‘change scores’ by Friedman’s method.

In lines 367-69 [Missing values of measurements were assigned the mean value of the group and time period [39]]. Please check the correctness/context of quoting reference 39 {MillerJ. Another warning about median reaction time. J Exp Psychol Hum Percept Perform. 1988; 14(3): 539–543}. There seems to be some confusion there.

What is ‘df’ in table-1 [**Table 1. Characteristics of the study participants**]? As stated in lines 354-55 {the primary and secondary outcomes at baseline were compared using the Mann–Whitney U test} ‘U’ stands for Mann–Whitney test statistic which does not have ‘df’, then this df corresponds with which test? Basically, I did not understand ‘why do you used so much statistics [df; U; p values, Shapiro–Wilk p; Levene p] here in table-1. In this context, I request authors to read the following {again pasted from the same textbook on ‘Medical Research Methodology’}:

To provide a description of baseline characteristics is entirely reasonable (since it is clearly important in assessing to whom the results of the trial can be applied), however, statistical comparison of baseline characteristics when random allocation/assignment is used/done [often for good/standard/leading journals these days] is not required, because even if P-value(s) turn(s) out to be significant (while comparing baseline characteristics despite random allocation), it is, by definition, a false positive as you then are supposed to be testing ‘randomization’ then, which in any single trial may not balance all baseline characteristics (particularly when sample sizes are small). Remember that ‘randomization’ is a sort of ‘insurance’ and not a guarantee scheme. Authors may please refer to following articles:

References:

1. Stuart J. Pocock, et al., ‘Subgroup analysis, covariate adjustment and baseline comparisons in clinical trial reporting: current practice and problems’, Statistics in medicine, 2002; 21:2917–2930 [Particularly page 2927]
2. Harrington D, et al., ‘New guidelines for statistical reporting in the journal’, N Engl J Med 2019;381:285-6

[Important message (indirectly/ultimately indicated) from these articles: Never do any comparison with respect to ‘baseline’ characteristics {by applying statistical significance test(s)}, when allocation is done randomly].

However, Statistical comparison [only with respect to important/indicated variables] of baseline characteristics may be performed, to find out if analysis adjustment (say stratified analyses or else) is required with respect to these variables.

You should justify all statements (give purpose etc.), clarify details and properly interpret all the results (P-values). How you analysed data of table-2,3,4 {**Table 2. Comparisons of gaps between conjectural and actual range of motions among groups at baseline, Table 3. Group comparisons for actual ROM, conjectural ROM, gap between actual and conjectural ROM, and PRWE score, Table 4. Comparisons of gaps between conjectural and actual range of motions between the study groups**} is not clear. Note that while reporting Confidence Interval’ it is preferable to use ‘to’ [instead of ‘-‘ or ‘,’] between two numerical values, so as to avoid confusion with respect to negative (-) sign, for example, in table-3: for VF_Gap	-13 (-28, -2) at Baseline, in AOT group, should be 95% CI: -28 to -2].

Because it is stated in lines 659 to 662 that “As this study was not a randomized controlled trial, the efficacy of this intervention must be verified in follow-up studies, including whether it is effective regardless of age and gender” and considering very small sample used for the study plus many other faults/limitations {example allocation – lines 217-222: The allocation was not randomized; 10 patients were assigned to the Non-AOT group from September 2013 to August 2014 and to the AOT group from January 2016 to October 2018 for adjustment of the experimental equipment (Ghost) depending on the time of the year}, best to call/classify this study as ‘PILOT’ and mention this in title.

Abstract of one article on guidelines for reporting non-randomised studies {Reeves BC, Gaus W. ‘Guidelines for reporting non-randomised studies’, Forsch Komplementarmed Klass Naturheilkd. 2004 Aug;11 Suppl 1:46-52. doi: 10.1159/000080576} reproduced below will hopefully give idea of ‘what is expected’:

Non-randomised studies (NRSs) are more susceptible to bias. The Consolidated Standards of Reporting Trials (CONSORT) statement was established to ensure that researchers report features of RCTs that must be considered when appraising their quality. CONSORT has improved the reporting of key information, highlighting missing key information for users. Researchers have a responsibility to report essential information that allows users to assess the susceptibility of NRS to selection, performance, detection and attrition bias. This paper considers criteria for reporting cohort studies: the rationale behind the CONSORT criteria for reporting of RCTs will be applied to cohort studies. Many of the criteria need no modification but application of others raise difficult issues for cohort studies, e.g.: description and standardisation of control and intervention treatments; description of the method of allocation; choice of prognostic factors to be collected; distinguishing between intended and provided treatments; collection of data on adverse and longterm outcomes; establishing a priori plans for analysis.

As you know (& is well-known) that while reporting [findings from randomised or non-randomised even] ‘Clinical Trial’ one should follow CONSORT guidelines. I request authors to kindly check/ensure that important items {like How sample size was determined (Item 7a), Random Sequence generation (Item 8a), Allocation concealment (Item 9), Blinding (Item 11a)} are included [since your article type is ‘Clinical Trial’, you are supposed to cover these items in the report]. Refer to lines 222-24: patients were assigned to the two groups alternately in the order of their prescriptions until the sample size was met is not a standard way of allocation (please note).

As pointed out in ‘important note’ above “This review pertains only to ‘statistical aspects’ of the study and so ‘clinical aspects’ should be assessed separately/independently [one should carefully consider/look at the clinical implications of the study]. In my opinion, to rescue this article (which seems quite difficult, if not impossible), large amount of re-vision (re-drafting) may be needed. However, please do not limit the revision only (with respect) to comments made here. More improvement is expected. ‘Major revision’ is recommended [in lieu of plain rejection, assuming that the respected editor would like to give chance to authors for improvement of the manuscript].
