# Supplementary material for: Action observation intervention using three-dimensional movies improves the usability of hands with distal radius fractures in daily life-A nonrandomized controlled trial in women
Source: PLoS One. 2024 Oct 18;19(10):e0294301. doi: 10.1371/journal.pone.0294301 (PMC11488734; doi:10.1371/journal.pone.0294301)
Supplement: Attachment — Submitted filename: sbm_04_Response_to_Reviewers_20240504h.docx [file pone.0294301.s009.docx]

May 4^th^, 2024

Hanna Landmark

Editor
*PLOS ONE*

Dear Dr. Hanna Landmark,

We would like to thank you and the reviewers for your careful review of our manuscript. The insightful statistical perspective of the reviewers offered invaluable suggestions that greatly enriched our work. As the first author, Kengo Usuki, is a novice researcher, your feedback has been particularly valuable in enhancing our understanding and approach. This study has proven to be a beneficial experience for us, thanks in large part to your contributions.

The manuscript has been rechecked and the necessary changes have been made in accordance with the reviewers’ suggestions. The responses to all comments have been prepared and given below. For your convenience, all changes in the revised manuscript are highlighted in yellow.

Thank you for your consideration. I look forward to hearing from you.

Sincerely,

Toyohiro Hamaguchi, Ph.D

Department of Rehabilitation,

Graduate School of Health Sciences,

Saitama Prefectural University,

820, Sannomiya, Koshigaya, Saitama, Japan

Phone: +81-48-971-4125

Fax: +81-48-973-4807

Email: hamaguchi-toyohiro@spu.ac.jp

**Response for reviewer 1**

**Reviewer 1, Comment＃1**

**I noted that your ABSTRACT is well drafted (in my opinion), but is ‘assay type’. It is preferable [refer to item 1b of CONSORT checklist 2010: Structured summary of trial design, methods, results, and conclusions] to divide the ABSTRACT with small sections like ‘Objective(s)’, ‘Methods’, ‘Results’, ‘Conclusions’, etc. which is an accepted practice of most of the good/standard journals [including this one, though ‘The PLoS One Guidelines to Authors’ did not specify an Abstract format, it is desirable].**

**Response to Comment #1**

As per your comment, we have added the “Objective,” “Trial design,” “Method,” “Results,” and “Conclusions” headings to the abstract in accordance with the CONSORT 2010 guidelines.

**Reviewer 1, Comment＃2**

**This trial/study is on very important topic but title of the study [“Action observation intervention using three-dimensional movies improves the usability of hands with distal radius fractures in daily life”] is little confusing. It gives the impression that authors are interested [their main focus is on] in ‘three-dimensional video during ROM exercises’ whereas in lines 25-28 it is clearly mentioned that ‘This nonrandomized controlled trial tested the hypothesis that AOT improves hand-use difficulties during activities of daily living in patients with DRF’. Action observation intervention is the main intervention [lines 30-31: The primary outcome was the difficulty in using the fractured hand and line 27: AOT improves hand-use difficulties] which is evaluated but administered using three-dimensional movies. You may think of changing the title.**

**Response to Comment #2**

As per your suggestion, we have revised the title to “Pilot study: Action observation intervention using three-dimensional movies improves the usability of hands with distal radius fractures in daily life: a nonrandomized controlled trial in women.”

**Reviewer 1, Comment＃3**

**According to table-2 on page 158 of Jacob Cohen’s paper “A power primer” in Psychological Bulletin, 1992, vol.:112, pp 155-159 [which is a sort of summary of the excellent book by Cohen himself titled ‘Statistical power analysis for the behavioral sciences’, Academic Press, 1977, New York] even for medium effect size you need n=64 per group (type-I error=0.05, power=80%). This is given here in the context of account in lines 335-36 [where you said: “the final target sample size was set to be 34 cases in total, 17 cases in each group”]. Although you have quoted the reference number 28 { Schott N, Korbus H. Preventing functional loss during immobilization after osteoporotic wrist fractures in elderly patients : a randomized clinical trial. BMC Musculoskelet Disord. 2014; 15: 287 but this study is a randomized with three intervention groups & as I understand, it is only a protocol} please check the ‘control’ group to be used and all other issues. Was it (study described in reference 28) simillar [with respect to ‘design’] to yours?**

**Response to Comment #3**

We agree that our sample size was small and the Patient Rated Wrist Evaluation (PRWE) scores did not have a normal distribution. Initially, we had considered using analysis of variance. Instead, PRWE, as reported in a previous study (Nadia Schott. 2014), used as the outcome. We believe that the designs are similar. In the future, we plan to continue collecting samples and further validate this finding by using variance analysis.

Reference:

Schott N, Korbus H. Preventing functional loss during immobilization after osteoporotic wrist fractures in elderly patients: A randomized clinical trial. BMC Musculoskelet Disord. 2014;15: 287.

**Reviewer 1, Comment＃4**

**Since (lines 340-41) “The primary outcome of this study was the PRWE score as a measure of the difficulty in using hands for ADLs” please check the level of measurement of data (to be) yielded by ‘The PRWE scale/questionnaire’. Though this measure/tool used is appropriate, is likely to yield data that are in [at the most] ‘ordinal’ level of measurement [and not in ratio level of measurement for sure {as the score two times higher does not indicate presence of that parameter/phenomenon as double (for example, a Visual Analogue Scales VAS score or say ‘depression’ score)}].** **Then application of suitable non-parametric (or distribution free) test(s) is/are indicated/advisable [even if distribution may be ‘Gaussian’ (also called ‘normal’)]. Agreed that there is/are no non-parametric test(s)/technique(s) available to be used as alternative in all situation (s), but should be used whenever/wherever they are available. Therefore, in short use suitable non-parametric test(s)/technique(s) while dealing with data that are in ‘ordinal’ level of measurement even if [despite that] the distribution may be ‘Gaussian’.**

**Response to Comment #4**

Thank you for your important point. We first conducted the Friedman test on the VR group. Similar to the generalized linear model (GLM) results, only PRWE showed a cautious difference, depending on the period (Table).

**Post hoc test: the Durbin–Conover test.**

**PRWE_Total**: A significant difference exists for baseline to 1 month after surgery (p<0.01), 1 month after surgery to 2 months after surgery (p<0.01), and 2 months after surgery to 3 months after surgery (p<0.01).

**PRWE_Pain**: A significant difference exists for baseline to 1 month after surgery (p=0.01), 1 month after surgery to 2 months after surgery (p=0.01), and 2 months after surgery to 3 months after surgery (p<0.01).

**PRWE_Specific**: A significant difference exists for baseline to 1 month after surgery (p<0.01), 1 month after surgery to 2 months after surgery (p<0.01), and 2 months after surgery to 3 months after surgery (p<0.01).

**PRWE_Usual**: A significant difference exists for baseline to 1 month after surgery (p<0.01) and 1 month after surgery to 2 months after surgery (p<0.01).

**Actual_ROM_Volar_Flexion**: A significant difference exists for baseline to 1 month after surgery (p<0.01) and 1 month after surgery to 2 months after surgery (p<0.01).

**Actual_ROM_Dorsal_Flexion**: A significant difference exists for baseline to 1 month after surgery (p<0.01), 1 month after surgery to 2 months after surgery (p<0.01), and 2 months after surgery to 3 months after surgery (p=0.04).

**Actual_ROM_Pronation**: A significant difference exists for baseline to 1 month after surgery (p<0.01) and 1 month after surgery to 2 months after surgery (p<0.01).

**Actual_ROM_Supination**: A significant difference exists for baseline to 1 month after surgery (p<0.01).

Among all terms in the post hoc test, no significant difference existed for Gap_Volar_Flexion, Gap_Dorsal_Flexion, Gap_pronation, and Gap Supination.

However, the Friedman test cannot be used to compare the VR group and the Control group. The results of GLM are similar to those of PRWE when comparing between groups, and we believe that this is close to the Friedman's test. GLM can specify the distribution of the response variables through link functions and probability distributions. This allows them to be applicable to various types of data that are not limited to normal distribution including binomial distribution, Poisson distribution, and negative binomial distribution. This means that the model can be customized according to the specific data type or research needs. Additionally, research papers have analyzed the values of the PRWE using ANOVA. (1, 2) Therefore, we have analyzed the data of this study by adopting GLM.

References:

1. Watson N, Haines T, Tran P, Keating JL. A comparison of the effect of one, three, or six weeks of immobilization on function and pain after open reduction and internal fixation of distal radial fractures in adults a randomized controlled trial. Journal of Bone and Joint Surgery - American Volume. 2018;100(13):1118–25.

2. Thorninger R, Romme KL, Wæver D, Henriksen MB, Tjørnild M, Lind M, et al. Posttraumatic arthritis and functional outcomes of nonoperatively treated distal radius fractures after 3 years. Sci Rep. 2023 Dec 1;13(1).

**Reviewer 1, Comment＃5**

**In lines 367-69 [Missing values of measurements were assigned the mean value of the group and time period [39]]. Please check the correctness/context of quoting reference 39 {MillerJ. Another warning about median reaction time. J Exp Psychol Hum Percept Perform. 1988; 14(3): 539–543}. There seems to be some confusion there.**

**Response to Comment #5**

Thank you for pointing this out. The text has been corrected. Please refer to line 223.

**Reviewer 1, Comment＃6**

**What is ‘df’ in table-1 [Table 1. Characteristics of the study participants]? As stated in lines 354-55 {the primary and secondary outcomes at baseline were compared using the Mann–Whitney U test} ‘U’ stands for Mann–Whitney test statistic which does not have ‘df’, then this df corresponds with which test? Basically, I did not understand ‘why do you used so much statistics [df; U; p values, Shapiro–Wilk p; Levene p] here in table-1. In this context, I request authors to read the following {again pasted from the same textbook on ‘Medical Research Methodology’}: To provide a description of baseline characteristics is entirely reasonable (since it is clearly important in assessing to whom the results of the trial can be applied), however,** **statistical comparison of baseline characteristics when random allocation/assignment is used/done [often for good/standard/leading journals these days] is not required, because even if P-value(s) turn(s) out to be significant (while comparing baseline characteristics despite random allocation), it is, by definition, a false positive as you then are supposed to be testing ‘randomization’ then, which in any single trial may not balance all baseline characteristics (particularly when sample sizes are small). Remember that ‘randomization’ is a sort of ‘insurance’ and not a guarantee scheme. Authors may please refer to following articles:**

**Response to Comment #6**

We acknowledge this comment. We have revised Table 1. With regard to the use of *df*, we initially believed its use was necessary, as with the *t*-test. However, upon reflection and considering the nature of our study as a nonrandomized controlled trial, we have concluded that it would be more appropriate to delineate normality and homoscedasticity as the primary rationale for our chosen analysis method. Therefore, *df* has been deleted and the Mann–Whitney *U* information has been retained. Details regarding normality and homoscedasticity remain within the main text for clarity and transparency.

**Reviewer 1, Comment #7**

**You should justify all statements (give purpose etc.), clarify details and properly interpret all the results (P-values). How you analysed data of table-2,3,4 {Table 2. Comparisons of gaps between conjectural and actual range of motions among groups at baseline, Table 3. Group comparisons for actual ROM, conjectural ROM, gap between actual and conjectural ROM, and PRWE score, Table 4. Comparisons of gaps between conjectural and actual range of motions between the study groups} is not clear. Note that while reporting Confidence Interval’ it is preferable to use ‘to’ [instead of ‘-‘ or ‘,’] between two numerical values, so as to avoid confusion with respect to negative (-) sign, for example, in table-3: for VF_Gap -13 (-28, -2) at Baseline, in AOT group, should be 95% CI: -28 to -2].**

**Response to Comment #7**

The purpose has been added to the explanations in Tables 2–4 in the main text (Table 2; Table 3; Table 4). The interpretation of the results has also been described. In Table 2 and Table 4, “Gaps between conjectural and actual range of motions” was initially written as “Difference between.” This text has been changed to “Gap between” In Table 3, the interpretation of the results has been provided.

**Reviewer 1, Comment＃8**

**Because it is stated in lines 659 to 662 that “As this study was not a randomized controlled trial, the efficacy of this intervention must be verified in follow-up studies, including whether it is effective regardless of age and gender” and considering very small sample used for the study plus many other faults/limitations {example allocation – lines 217-222: The allocation was not randomized; 10 patients were assigned to the Non-AOT group from September 2013 to August 2014 and to the AOT group from January 2016 to October 2018 for adjustment of the experimental equipment (Ghost) depending on the time of the year}, best to call/classify this study as ‘PILOT’ and mention this in title.**

**Response to Comment #8**

We have added the term “Pilot study” to the title.

**Reviewer 1, Comment＃9 & 10**

**Abstract of one article on guidelines for reporting non-randomised studies {Reeves BC, Gaus W. ‘Guidelines for reporting non-randomised studies’, Forsch Komplementarmed Klass Naturheilkd. 2004 Aug;11 Suppl 1:46-52. doi: 10.1159/000080576} reproduced below will hopefully give idea of ‘what is expected’: Non-randomised studies (NRSs) are more susceptible to bias. The Consolidated Standards of Reporting Trials (CONSORT) statement was established to ensure that researchers report features of RCTs that must be considered when appraising their quality. CONSORT has improved the reporting of key information, highlighting missing key information for users. Researchers have a responsibility to report essential information that allows users to assess the susceptibility of NRS to selection, performance, detection and attrition bias. This paper considers criteria for reporting cohort studies: the rationale behind the CONSORT criteria for reporting of RCTs will be applied to cohort studies. Many of the criteria need no modification but application of others raise difficult issues for cohort studies, e.g.: description and standardisation of control and intervention treatments; description of the method of allocation; choice of prognostic factors to be collected; distinguishing between intended and provided treatments; collection of data on adverse and longterm outcomes; establishing a priori plans for analysis.**

**As you know (& is well-known) that while reporting [findings from randomised or non-randomised even] ‘Clinical Trial’ one should follow CONSORT guidelines. I request authors to kindly check/ensure that important items {like How sample size was determined (Item 7a), Random Sequence generation (Item 8a), Allocation concealment (Item 9), Blinding (Item 11a)} are included [since your article type is ‘Clinical Trial’, you are supposed to cover these items in the report]. Refer to lines 222-24: patients were assigned to the two groups alternately in the order of their prescriptions until the sample size was met is not a standard way of allocation (please note). As pointed out in ‘important note’ above “This review pertains only to ‘statistical aspects’ of the study and so ‘clinical aspects’ should be assessed separately/independently [one should carefully consider/look at the clinical implications of the study]. In my opinion, to rescue this article (which seems quite difficult, if not impossible), large amount of re-vision (re-drafting) may be needed. However, please do not limit the revision only (with respect) to comments made here. More improvement is expected. ‘Major revision’ is recommended [in lieu of plain rejection, assuming that the respected editor would like to give chance to authors for improvement of the manuscript].**

**Response to Comment # 9 & 10:**
We deeply appreciate the reviewer's guidance on the importance of adhering to the CONSORT guidelines for non-randomized studies, as outlined by Reeves BC and Gaus W. In response to the concerns raised, we have revised our manuscript to ensure that essential elements, as per the CONSORT extension for non-randomized trials, are comprehensively addressed. Here are the specific updates and justifications added to our manuscript:

Sample Size Determination (Item 7a): We have included a detailed description of how the sample size was determined in the revised manuscript. The rationale, based on preliminary data and statistical power analysis, is now thoroughly explained in section [Sample size calculation], lines 194-203. This modification provides readers with a clear understanding of the basis for our sample size, acknowledging the limitations and the study's statistical power. We acknowledge that our initial sample size of 34 cases (17 per group) is below the recommended n=64 per group for medium effect sizes, a decision influenced by the feasibility constraints within our study context. This explanation is now clearly detailed in our Methods section.

Random Sequence Generation and Allocation Concealment (Items 8a and 9): Considering our study's non-randomized nature, we have clarified the absence of random sequence generation and allocation concealment. However, to mitigate selection bias and emulate randomized study conditions as closely as possible, we employed an alternate assignment method, detailed in section [Experimental procedure], lines 117-123. We have also included a justification for the absence of random sequence generation, citing similar methodologies in studies like Schott and Korbus (2014), and discussed its potential impact on our findings. Finally, we have discussed the potential biases this method may introduce and how we have attempted to minimize them.

Blinding (Item 11a): We acknowledged in the manuscript that blinding of participants and researchers was not feasible in our study design due to the nature of the interventions. This limitation and its potential impact on the study's outcomes have been discussed in depth in section [Study limitation], lines 424-425, along with measures taken to reduce bias in outcome assessment.

Enhancing Reporting Transparency: We have revised our manuscript to include a CONSORT checklist at submission, ensuring that all relevant items are explicitly addressed or explained if not applicable. This checklist will facilitate a clear appraisal of the study's methodology and findings by readers and reviewers alike. We plan to continue our research, aiming to recruit additional participants to reach a more statistically powered sample size. This ongoing effort and its potential to validate our preliminary findings are now discussed in the study limitation section of our manuscript. Please refer to lines 444-446.

We believe these revisions comprehensively address the essential aspects of reporting non-randomized studies as highlighted by the reviewer.

**Response for reviewer 2**

**Reviewer 2, Comment＃1**

**The authors report a non-randomized study assessing the effect of virtual rehabilitation vs standard care after DRF. The authors perform a lot of measurements and find some faster recovery before 12 weeks after surgery.
A lot of data was collected. The main problem is, in my practice I don't send people generally to hand therapy after DRF. Several RCTs have shown that there is no difference, or people do better without. So I'm not sure what this virtual rehab truly adds to clinical practice.**

**Response to comment #1**

Thank you for your thought-provoking points. The cognitive and psychosocial aspects of musculoskeletal diseases, including the pain causalgia scale, constitute my main research themes. While hand therapy may not be imperative for cases showing progress, we contend that it is indispensable for instances experiencing setbacks and entering a state of stagnation. Although the present research theme does not directly relate to clinical practice, we identified that AOT has a positive effect on distal radius fractures. Furthermore, AOT can be performed using an assistive device and thus can be used for self-training at home. Hence, we anticipate its utility in aiding recovery.

Biopsychosocial aspects are crucial, but they are influenced by many factors that make them difficult to standardize. Clarifying the types of behaviors that patients tend to engage in after distal radius fractures and how specific exercises such as AOT can help can provide beneficial findings for distal radius fracture care. Please find below our detailed point-by-point responses to your individual comments.

**Reviewer 2, Comment＃2**

**Introduction
First 2 paragraphs can be omitted, or shortened into a few sentences.**

**Response to comment #2**

The text has been shortened to summarize that distal radius fractures occur more frequently in active women than other fractures, and to describe the necessity for women to quickly return to activities of daily living. Please refer to lines 44-48.

**Reviewer 2, Comment #3
Line 75-78: seems more like the authors opinion than fact. People generally find their own path of recovery, and some recover faster than others. I don’t think you can set goals like that. Some people are back to housework much faster than 7-8 weeks. You don’t want to slow them down with these goals.
Several randomized studies show no difference between people ‘rehabilitating’ with a hand therapist vs on their own. Some studies show people who do it on their own recover faster. Please include this in your introduction and discussion.
People can start some weightbearing activities right after volar plate fixation. I have people start finger motion right away, and wrist motion after 2 weeks. There are great early active rehab protocols out there.**

**Response to comment #3**

After consulting the orthopedic surgeon again, we reconfirmed the validity of our preliminary research. We are aware that there is no difference in the final outcome between therapy and self-training, with neither method posing a problem in the context of surgery. However, we would like to emphasize there are reports demonstrating good results with our initial protocol.

Previous research has shown that performing ADLs early on is better than delaying them (3,4). These outcomes were described in the manuscript: “Rehabilitation following distal radius fracture surgery aims to restore hand usability early. However, it has been reported that there is no difference in the final outcome between rehabilitation and voluntary training.” (lines 47-49)

In a previous study, it was reported that the volar locking plate was destroyed at 323N-673N in a clinical destructive test using a cadaver model (5–7). Additionally, in general, 250N is applied when exerting grip strength, and 400N is applied when using crutches. Clinically, the extent to which daily activities are permitted varies depending on the extent of osteoporosis and the surgical situation. This information was described in the paper: “However, regaining the ability to use the fractured hand for activities of daily living (ADLs) takes time, typically around 9 weeks for bone union post-surgery. Although early loading after volar locking plate surgery is possible, patients commonly experience difficulties in hand use for ADLs at 8 weeks postoperatively, with a reduction in these difficulties by 50% at 12 weeks.” (lines 51-55). We believe that the fixation strength is insufficient and can pose a risk, as there an objective issue of daily life not having been fully recovered in the early stage.

References:

3. Reid SA, Andersen JM, Vicenzino B. Adding mobilisation with movement to exercise and advice hastens the improvement in range, pain and function after non-operative cast immobilisation for distal radius fracture: a multicentre, randomised trial. J Physiother. 2020; 66(2):105–12.

4. Handoll HHG, Elliott J. Rehabilitation for distal radial fractures in adults. Vol. 2015, Cochrane Database of Systematic Reviews. John Wiley and Sons Ltd; 2015.

5. Liu X, Wu WD, Fang YF, Zhang MC, Huang WH. Biomechanical comparison of osteoporotic distal radius fractures fixed by distal locking screws with different length. PLoS One. 2014;9(7).

6. Huang JI, Peterson B, Bellevue K, Lee N, Smith S, Herfat S. Biomechanical Assessment of the Dorsal Spanning Bridge Plate in Distal Radius Fracture Fixation: Implications for Immediate Weight-Bearing. Hand (N Y). 2018;13(3):336.

7. Wall LB, Brodt MD, Silva MJ, Boyer MI, Calfee RP. The effects of screw length on stability of simulated osteoporotic distal radius fractures fixed with volar locking plates. Journal of Hand Surgery. 2012;37(3):446–53.

**Reviewer 2, Comment＃4**

**Internal and external rotation, you mean pronation and supination?**

**Response to comment #4**

Thank you for pointing this out. We have corrected it. Please refer to line 58.

**Reviewer 2, Comment #5**
**You state: “Action observation therapy (AOT) is an effective means of preventing DDP in patients”, that remains to be tested no?**

**Response to comment #5**
Thank you for pointing this out. Previous studies have reported that AOT reduces atrophy of the brain cortex, but this has not yet been reported in clinical practice for DRF, as you have stated. We believe this study will verify this. We have changed in manuscript to “If DDP occurs in patients with DRF and their ADLs are restricted by perceived difficulties in using their hands, then training to correct this phenomenon should be considered. Action observation therapy (AOT) may prevent DDP in patients.” (lines 79-81)

**Reviewer 2, Comment #6**

I think the introduction can be shortened.

**Response to comment #6**

We have done our best to reduce the word count of the introduction, which now stands at 580 words. Kindly let us know if further reduction is required. Please refer to lines 44-90.

**Reviewer 2, Comment #7**

**I think we need more information or a video on the exact Ghost program.**

**Response to comment #7**

We agree with this comment and have provided you with the video materials used for OSF. We have modified the main text “Data and study protocol availability statement” to reflect this. Please refer to line 464.

**Reviewer 2, Comment #8**
**Results
You exclude a seemingly large amount of data based on outliers. Can you mention a sensitivity analysis with those outliers? Are results the same?**

**Response to comment #8**

Thank you for your insightful comments. We have conducted a sensitivity analysis including the previously identified outliers. This analysis revealed that when outliers were included in the data set, there were no statistically significant differences in PRWE Total and Usual scores between the AOT and Non-AOT groups. However, the PRWE Specific scores retained a significant difference, suggesting that the specific interventions in the AOT may still have a targeted therapeutic effect not evident in the more general measures. These results underscore the importance of considering outliers in the analysis of clinical data, as they can influence the outcomes significantly.

To address this and provide a comprehensive understanding of our study's implications, we have revised the manuscript to include these findings: “PRWE Specific (R^2^=0.67, AIC=1102, χ^2^=14.28, p<0.01) showed a significant interaction between group and time.　No interaction existed between group and time for PRWE Pain (R^2^=0.43, AIC=1025, χ^2^=2.25, p=0.52), PRWE Usual (R^2^=0.57, AIC=1011, χ^2^=6.77, p=0.08), and PRWE Total (R^2^=0.65, AIC=1179, χ^2^=6.95, p=0.07)”. Please refer to 327-331.

"In sensitivity analyses that included data outliers, we found that the significant differences in PRWE Total and Usual scores previously observed were no longer present. This suggests that our earlier results might be sensitive to extreme values. However, the significant difference in PRWE Specific scores remained, indicating a persistent effect of the AOT intervention on specific functional improvements. These findings have been discussed in the context of their implications for clinical practice, particularly in settings where outlier data cannot be readily excluded (8,9)." (lines 414-422)

References:

8. Bakker M, Wicherts JM. Outlier removal and the relation with reporting errors and quality of psychological research. PLoS One. 2014 Jul 29;9(7).

9. Pollet T V., van der Meij L. To Remove or not to Remove: the Impact of Outlier Handling on Significance Testing in Testosterone Data. Adaptive Human Behavior and Physiology. 2017 Mar 1;3(1):43–60.

**Reviewer 2, Comment #9
Consider grouping some outcomes. If you take every ROM separately, you are performing a lot of analysis. Why not use total ROM for example as a single outcome?**

**Response to comment #9**

As you pointed out, repeating the analysis might introduce more errors, but unlike with fingers and fingers, if we group the ROMs together, we will not be able to distinguish the parts which have improved. Hence, we analyzed them individually.

**Reviewer 2, Comment #10**

**Discussion
Is all of this worth a slight improvement at 4 weeks, while at 3 months there isn’t much of a difference? This is not a RCT. Keep in mind that previous RCTs show no benefit to hand therapy after DRF in most people. Generally the bio-psycho-social model of health is preferred. I would discuss the role of mental health and ROM/PRWE after distal radius fracture. Some studies show it explains 70% of the variation in recovery. Your focus on motor cortex brain blood flow is too limited, it’s only the bio part of health, without the psycho-social which seems to be more important.**

**Response to comment #10**

We agree with your point. We have reviewed the literature again and reported that there was no difference between the self-training group and the no-training group after volar locking plate surgery (4). Regarding therapy, similar to the current study, it was reported that PRWE improved consciously in the voluntary training group and rehabilitation group after 4 weeks (3). We believe that this has a certain clinical significance. Although a biopsychosocial model is important (10,11), we will not consider it here.

We have added the following sentences to the discussion in the main text. “Previous research has suggested that a biosocial psychological model is involved in recovery after distal radius fracture surgery (10,11). Steaven et al. state that high or increasing levels of catastrophizing had an increased risk for a less than full recovery of strength by almost six-fold. It is unclear what characteristics the participants had in terms of biosocial psychology, and what kind of influence AOT had on them.” (lines 409-413)

References:

3.	Reid SA, Andersen JM, Vicenzino B. Adding mobilisation with movement to exercise and advice hastens the improvement in range, pain and function after non-operative cast immobilisation for distal radius fracture: a multicentre, randomised trial. J Physiother. 2020; 66(2):105–12.

4. Handoll HHG, Elliott J. Rehabilitation for distal radial fractures in adults. Vol. 2015, Cochrane Database of Systematic Reviews. John Wiley and Sons Ltd; 2015.

10.	Collis JM, Mayland EC, Wright-St Clair V, Signal N. “The more I do, the more I can do”: perspectives on how performing daily activities and occupations influences recovery after surgical repair of a distal radius fracture. Disabil Rehabil. 2022;44(19):5440–9.

11. Steaven J, Nina B. Pain-related fear, catastorophizing and Pain in the recovery from a fracture. Scandinavian Journal of Pain. 2010;1: 38-42

**Reviewer 2, Comment＃11
“It has been reported that the flexor digitorum longus tendon, which is elongated during dorsiflexion, is impaired after volar locking plate surgery”. I don’t know which tendon this is, and I am also not sure the muscle is impaired. Possibly FPL, but that should limit ROM too much.**

**Response to comment #11**

It is the flexor pollicis longus. This has been corrected. Please refer to line 379.

The meaning of this sentence does not mean a direct rupture, but rather that the impact is caused by soft tissue adhesions. (lines 380-382)

**Reviewer 2, Comment #12
“square iliacus muscle”, do you mean pronator quadratus?**

**Response to comment #12**

Thank you for your advice. It was a typo. The name has been corrected. Please refer to line 381.

**Reviewer 2, Comment #13
It’s a bit unclear to me how the virtual model truly helps increase motion.**

**Response to comment #13**

We believe that in this study, we found the possibility that AOT is effective. Hence, we agree that this study alone is insufficient to show how the virtual model helps increase motion, and that future studies must perform further analyses and increase the number of included cases.

**Response for reviewer 3**

**Reviewer 3, Comment #1**

**This study aims to compare two different types of postoperative physical therapy after surgery for distal radius fractures. The study spanned eight years and included 35 patients, all of whom were female and had fractures in the right hand, their dominant hand. This indicates that the study rigorously controlled for various factors that could influence surgical outcomes. The study showed patients who underwent AOT had better PRWE scores in weeks 4 and 8. Such results are not very surprising, mainly because the comparison depends on the control group (non-AOT) and the postoperative rehabilitation protocol (unfortunately, not described in this study). Many past studies have already told us that early mobilization helps with short-term outcomes, but there won't be much difference in intermediate-term results, which this study confirms.**

**Response to comment #1**

Thank you for your thought-provoking comments. Although we were unable to conduct an RCT, we did include control patients. The intervention method is as described in the Intervention method part, and we followed the protocols of Hoopenfeld et al. (12). However, the protocol may differ slightly depending on the individual circumstances of the patient and the therapist in charge. In this study, there was a short-term effect, but ultimately no difference was observed. We believe that distal radius fractures may initially improve the perception of hand-use difficulty during ADL. We intend to further investigate this phenomenon.

Reference:

12. Hoppenfeld S, Murthy VL. Treatment and rehabilitation of fractures. Bronx, NY: Lippincott Williams and Wilkins; 2000; 191-206.

**Reviewer 3, Comment #2**

**Introduction:
1. Please shorten the introductory paragraph to under 500 words, providing sufficient background knowledge and detailing the clinical dilemma faced by the surgeon.**

**Response to comment #2**

To the best of our ability, we have reduced the word count of the introduction, which is now approximately 580 words. Please refer to the Introduction section, lines 44-90.

**Reviewer 3, Comment #3**

**2. Lines 90-100 and 118-119: Please replace Internal Rotation/External Rotation with pronation/supination.
Response to comment #3**

The name has been corrected. Please refer to line 58.

**Reviewer 3, Comment #4**

**3. Line 105: It is quite strange that Figure 2 is placed here.
4. Line 178: Where is Figure 3?**

**Response to comment #4**

Thank you for your advice. We have corrected the figure number.

**Reviewer 3, Comment＃5**
**5. Lines 176-178, please pose a clear question this manuscript seeks to answer: Can AOT in the early postoperative period of a distal radius fracture (DRF) promote quicker functional recovery? What are the primary and secondary outcomes for measuring its effectiveness?**

**Response to comment #5**
The hypothesis of this study is as stated in the main text: “This study tested the hypotheses that AOT in the early postoperative period of DRF can (1) prevent the underestimation of ROM, (2) attenuate hand-use difficulty in ADL, and (3) improve joint ROM early in patients with DRF.” (Lines 88-90)

We decided the main outcome for the study was PRWE, and the secondary were actual and estimated ROM. Please refer to lines 131-132.

**Reviewer 3, Comment #6**
**Methods:
1. This study was not randomized, and the number of participants was small. Please describe the fracture patterns of the distal radius fractures included in the study, whether only volar locking plates were used, or whether any patients underwent additional plating for specific fragment fixation.**

**2. Compared to AOT, does the non-AOT group follow a specific standardized protocol?**

**3. Was bony consolidation confirmed by X-rays after surgery? Was there any difference between the two groups?**

**Response to comment #6**

As per your instructions, we have reviewed the medical records. Compared to AOT, the non-AOT group followed the intervention method described in the manuscript and post-therapy was performed according to a standardized protocol (12). Two patients in the AOT group and seven patients in the Non-AOT group underwent surgery using fixation materials such as a long plate, percutaneous pinning, and an ulnar plate other than the usual volar locking plate. At the time of evaluation, one patient in the AOT group and two patients in the Non-AOT group were diagnosed as having not achieved complete bone union. Many patients received fixation materials other than locking plate fixation, and there was one non-AOT patient who had incomplete bone union at the 3-month evaluation, which may have affected the results of surgery. Aspects of the surgical procedure and delayed bone union likely influenced the results. The above contents are described in the results and research limitations, as follows;

“Two patients in the AOT group and seven patients in the Non-AOT group underwent surgery using fixation materials such as a long plate, percutaneous pinning, and an ulnar plate other than the usual volar locking plate. At the time of evaluation, one patient in the AOT group and two patients in the Non-AOT group were diagnosed as having not achieved complete bone union.” (lines 239-243)

“Compared with the AOT group, more patients in the Non-AOT group received fixation materials other than volar locking plate fixation. Additionally, there was one non-AOT patient who was considered to have incomplete bone union at the 3-month evaluation, which may have affected the results. The surgical procedure and the delay in bone union are likely to have influenced the results.” (lines 428-432)

Reference:

12. Hoppenfeld S, Murthy VL. Treatment and rehabilitation of fractures. Bronx, NY: Lippincott Williams and Wilkins; 2000; 191-206.

**Reviewer 3, Comment #7
Results:
1. Figure legends scattered irregularly throughout the manuscript**

**Response to comment #7**

Thank you for your advice. The number and legends in the diagram have been corrected. The location of legends in the text follows the instructions of PLoS One. We follow this regulation.

**Reviewer 3, Comment＃8**

**2. The presentation of tables is very chaotic, difficult to read the figures.**

**Response to comment #5**

We understood this comment. We have modified Tables.

**Reviewer 3, Comment #9**

**Discussion:
1. Line 582: What is the square iliacus muscle?**

**Response to comment #9**
It was a mistake in the pronator quadratus muscle. We have corrected this term. (line 381)

**Reviewer 3, Comment #10**
**2. Please discuss why there is no difference between the two groups in 12 weeks.**

**Response to comment #10**

Thank you for your observation regarding the absence of significant differences between the AOT and Non-AOT groups at the 12-week assessment. This finding warrants careful consideration in light of the pathophysiological recovery process and the nature of our intervention.

Firstly, it is important to recognize that distal radius fractures typically show a substantial degree of natural healing that progresses over the weeks following injury. As reported in studies such as Kristiansen et al. (1997) (13), most patients experience significant improvements in function and pain within the first 8 to 10 weeks post-fracture due to natural healing processes, regardless of the specific rehabilitation approach employed. Furthermore, by 12 weeks, most patients have reached a stage of recovery where differences in early intervention effects may become less pronounced. This phenomenon has been documented in the literature, where the initial beneficial effects of specific therapies tend to converge with outcomes of standard treatment over time as the natural course of healing overshadows initial therapeutic benefits. This is supported by MacDermid et al. (2003) (14), who observed that improvements in function and pain after distal radius fractures tend to plateau after the initial recovery period. Additionally, the action observation therapy (AOT) employed in our study primarily aims to enhance motor imagery and early mobilization. Rocca et al. (2017) (15)have suggested that while AOT can significantly influence cortical reorganization and early functional recovery, its long-term effects might not differ significantly from conventional therapy as patients approach clinical equilibrium. Moreover, by 12 weeks, other factors such as individual variations in rehabilitation adherence, psychological factors, and personal motivation might play an increasingly significant role, potentially diluting the measurable impact of AOT compared to usual care. The potential for such confounding factors has been discussed by Opie et al. (2016) (16), who highlighted how patient-specific factors could impact the efficacy of rehabilitation interventions over longer periods. Thus, the absence of differences between the AOT and Non-AOT groups at 12 weeks can be attributed to the substantial natural recovery observed by this stage, the potential plateauing of benefits from specific interventions like AOT, and the increasing influence of individual patient factors on recovery outcomes. For future studies, continuing to monitor outcomes beyond this point could provide further insights into the long-term efficacy of AOT and its potential role in sustaining improvements beyond the natural recovery timeline.

References:

13.	Kristiansen TK, Ryaby JP, McCabe J, Frey JJ, Roe LR. Accelerated healing of distal radial fractures with the use of specific, low-intensity ultrasound. A multicenter, prospective, randomized, double-blind, placebo-controlled study. J Bone Joint Surg Am. 1997;79(7):961–73.

14.	MacDermid JC, Roth JH, Richards RS. Pain and disability reported in the year following a distal radius fracture: a cohort study. BMC Musculoskelet Disord. 2003;4:1–13.

15.	Rocca MA, Fumagalli S, Pagani E, Gatti R, Riccitelli GC, Preziosa P, et al. Action observation training modifies brain gray matter structure in healthy adult individuals. Brain Imaging Behav. 2017;11(5):1343–52.

16.	Opie GM, Evans A, Ridding MC, Semmler JG. Short-term immobilization influences use-dependent cortical plasticity and fine motor performance. Neuroscience. 2016;330:247–56.
