# Supplementary material for: Action observation intervention using three-dimensional movies improves the usability of hands with distal radius fractures in daily life-A nonrandomized controlled trial in women
Source: PLoS One. 2024 Oct 18;19(10):e0294301. doi: 10.1371/journal.pone.0294301 (PMC11488734; doi:10.1371/journal.pone.0294301)
Supplement: Attachment — Submitted filename: sbm_05_Response_to_Reviewers_20240531_hh.docx [file pone.0294301.s011.docx]

**Response for reviewer 1**

***Reviewer #1: COMMENTS: Since most of the comments made on earlier draft are considered positively & attended, I recommend the acceptance after very minor revision. The manuscript now has achieved acceptable level, in my opinion (just need to mention few things/limitations taking clue from following). As the study is now labelled ‘pilot’ in nature, sample size [and/or rigorous methodology] is/are not a big issue.***

***However, it is very important to note that ‘RANDOMIZATION’ described in lines 117-123 [Experimental procedures: Study participants were assigned to one of two groups: the group that received AOT and the group that received the usual rehabilitation without AOT (Non-AOT). The allocation was not randomized: 10 patients were assigned to the Non-AOT group from September 2013 to August 2014 and to the AOT group from January 2016 to October 2018 to adjust the experimental equipment (i.e., Ghost), depending on the time of the year. The patients were thereafter assigned to the two groups alternately in the order of their prescriptions until the sample size was met in March 2021.] is not scientific and so not agreeable. But since the study is now labelled ‘pilot’ in nature, randomization described is not that important/vital and so can be ignored/accepted.***

***Also note that the responses to all the comments (by all reviewers) are/were with unnecessary details and/or references {on many occasions}. They are not ‘precise’ as desired. At times very lengthy.***

**Response to Comment #1**

Thank you for your insightful comments and for acknowledging the improvements made in the manuscript. We appreciate your recommendation for acceptance after minor revisions and agree that this pilot study's nature makes the sample size and rigorous methodology less critical.

We understand that true randomization is the gold standard for reducing bias and increasing the robustness of study results. However, given the nature of the pilot for this study, we believe that this approach was the only way to achieve the exploratory objectives of this initial survey. We acknowledge this as a limitation and recognize that true randomization would enhance the robustness of the findings. Nevertheless, the pilot nature of this study aims to provide preliminary insights that could inform the design of future randomized controlled trials.

Additionally, we take feedback on our responses to reviewers' comments very seriously. We have reviewed and revised our manuscript to ensure that our responses were concise and accurate, focusing on directly addressing the reviewers' concerns without including unnecessary details or references.

Thank you once again for your valuable feedback, which has significantly contributed to the improvement of our manuscript.
